# Biofeedback improves postural control recovery from multi-axis discrete perturbations

**DOI:** 10.1186/1743-0003-9-53

**Published:** 2012-08-03

**Authors:** Kathleen H Sienko, M David Balkwill, Conrad Wall

**Affiliations:** 1 Massachusetts Institute of Technology, Cambridge, MA, USA; 2 Jenks Vestibular Diagnostic Laboratory, Massachusetts Eye and Ear Infirmary, Boston, MA, USA; 3 Department of Otology & Laryngology, Harvard Medical School, Boston, MA, USA; 4 Department of Mechanical Engineering, University of Michigan, Ann Arbor, MI, USA; 5 Department of Biomedical Engineering, University of Michigan, Ann Arbor, MI, USA

**Keywords:** Vibrotactile, Biofeedback, Balance, Perturbations, Intuitive display, Sensory augmentation, Sensory substitution, Vestibular

## Abstract

**Background:**

Multi-axis vibrotactile feedback has been shown to significantly reduce the root-mean-square (RMS) sway, elliptical fits to sway trajectory area, and the time spent outside of the no feedback zone in individuals with vestibular deficits during continuous multidirectional support surface perturbations. The purpose of this study was to examine the effect of multidirectional vibrotactile biofeedback on postural stability during discrete multidirectional support surface perturbations.

**Methods:**

The vibrotactile biofeedback device mapped tilt estimates onto the torso using a 3-row by 16-column tactor array. The number of columns displayed was varied to determine the effect of spatial resolution upon subject response. Torso kinematics and center of pressure data were measured in six subjects with vestibular deficits. Transient and steady state postural responses with and without feedback were characterized in response to eight perturbation directions. Four feedback conditions in addition to the tactors off (no feedback) configuration were evaluated. Postural response data captured by both a force plate and an inertial measurement unit worn on the torso were partitioned into three distinct phases: ballistic, recovery, and steady state.

**Results:**

The results suggest that feedback has minimal effects during the ballistic phase (body’s outbound trajectory in response to the perturbation), and the greatest effects during the recovery (return toward baseline) and steady state (post-recovery) phases. Specifically, feedback significantly decreases the time required for the body tilt to return to baseline values and significantly increases the velocity of the body’s return to baseline values. Furthermore, feedback significantly decreases root mean square roll and pitch sway and significantly increases the amount of time spent in the no feedback zone. All four feedback conditions produced comparable performance improvements. Incidences of delayed and uncontrolled responses were significantly reduced with feedback while erroneous (sham) feedback resulted in poorer performance when compared with the no feedback condition.

**Conclusions:**

The results show that among the displays evaluated in this study, no one tactor column configuration was optimal for standing tasks involving discrete surface perturbations. Feedback produced larger effects on body tilt versus center of pressure parameters. Furthermore, the subjects’ performance worsened when erroneous feedback was provided, suggesting that vibrotactile stimulation applied to the torso is actively processed and acted upon rather than being responsible for simply triggering a stiffening response.

## Background

Sensory augmentation is a technique for supplementing native sensory inputs. In the context of balance applications, it provides users with additional cues about body motion, usually with respect to a gravito-inertial environment. Typical sensory augmentation systems comprise a motion or force sensor to detect body kinematics or kinetics, respectively; a processor to estimate body kinematics or center of pressure; and a feedback display to provide the user with an additional channel of information. Vibrotactile [1], electrotacile [2], visual [3], auditory [4], and multi-modal [5,6] feedback systems are currently being investigated for their utility to serve both as a real-time balance aid for individuals with sensory loss and older adults, as well as a balance rehabilitation training tool. Although electrotactile, visual, and auditory displays are all valid and effective means of conveying spatial orientation information, sensory augmentation in the form of a vibrotactile display is preferential because vibrotactile stimulation does not compete with tasks that involve speaking, eating, seeing, and hearing [7,8].

Torso-based vibrotactile displays convey information to the user in an intuitive fashion since stimuli are directly mapped to the body coordinates (e.g. left is left, front is front, etc.) [9]. Cholewiak et al. showed that the ability to localize vibratory stimuli is a function of separation among loci and location on the torso; specifically, anatomically defined anchor points at the navel and spine enhance performance when the spatial resolution of the display is decreased [10]. Therefore, torso-based vibrotactile displays are good candidates for use in displaying body tilt during standing and locomotor activities. However, questions remain about the best way to use vibrotactile displays to code magnitude and direction of body motion to the user. In a design study examining vibrotactile display coding, performance in a modified version of the manual control critical tracking task was not appreciably improved when more than three rows of position-based tactors were used [11]. Circumferential spatial resolution becomes an issue when providing multidirectional tilt information. An argument can be made for having the greatest spatial resolution allowable by two-point discrimination in order to supply the operator with the maximum amount of information regarding his/her tilt. On the other hand, there is the issue of cognitive load: the more information that is provided to the user, the more potentially taxing it is to interpret and use that information. In this study, we varied the spatial resolution of the feedback while giving subjects discrete support surface perturbations while standing.

Postural perturbations are commonly achieved in the clinical or laboratory setting by continuous and discrete translations and rotations of the support surface. However, standard perturbation-based systems such as computerized dynamic posturography [12] are limited to single-axis dynamics and therefore the majority of perturbation-based assessments of feedback systems have been performed along the sagittal axis (referred to here as anterior-posterior (A/P)). Real-time vibrotactile feedback of torso and head kinematics has been effective in improving postural stability in subjects with vestibular deficits during computerized dynamic posturography [13-15].

While previous studies have compared postural sway responses with and without vibrotactile feedback [14,15], these studies did not investigate the case of discrete perturbations given in unpredictable directions. This case is significant because it occurs in “real life,” for example while standing on a bus or subway car that is starting or stopping. Our previous study of spatial resolution of vibrotactile feedback while subjects stood on a continuously moving platform suggested that fine resolution was not crucial for good postural control since a spatial resolution of 90° was as effective as a spatial resolution of 22.5° [16,17].

The purpose of this study is fourfold: first, to determine the effect of torso-based vibrotactile feedback on postural performance as a function of multidirectional discrete support surface perturbations; second, to examine the effect of display spatial resolution on performance as a function of perturbation direction; third, to ascertain the periods within the response trajectory where feedback is most useful; and fourth, to determine the effect of erroneous (sham) feedback, in which the feedback signal did not reflect the subject’s actual body motion, on performance. We hypothesized that feedback would not significantly affect subjects’ postural response to the perturbation during the initial body sway away from the vertical (ballistic phase), but would quicken the return to upright stance (recovery phase) and improve standing balance following recovery (steady state phase). Based on the findings from the abovementioned continuous perturbation study, we hypothesized that performance would not be affected by spatial resolution.

## Methods

### Participants

Six subjects (5 males, 1 female, 47.8 ± 9.5 yrs) with vestibular deficits volunteered for this study, and had previously participated in the continuous perturbation study [16]. All subjects failed the NeuroCom^TM^ EquiTest^TM^ computerized dynamic posturography Sensory Organization Tests (SOT) 5 and 6. Exclusion criteria included any self-reported neurological impairments and failing scores on the Motor Control Test (MCT). Table 1 shows the subjects’ relevant demographics, SOT, MCT, and vestibular test results. Informed consent was obtained from each subject. The participating universities’ research ethics boards approved this study, which conformed to the Helsinki Declaration.

**Table 1:** Subject demographics and vestibular diagnoses

**Subject Demographics**			**Computerized Dynamic Posturography**				**Classification**		**Rotation Test**		**Caloric Test**	
**Subject ID**	**Age**	**Gender**	**SOT Score**	**SOT5**	**SOT6**	**MCT Score**	UVH or (pBVH)*	**Probability of normal VOR**	**VOR gain**	**Time Constant(s)**	**RVR (%)**	**Caloric Sum (°/s)**
1	55	M	49	Fall, Fall, Fall	Fall, Fall, Fall	N/A	BVH†	< .001	0.333	N/A	−100	3
2	45	M	45	Fall, Fall, Fall	Fall, Fall, Fall	128	(p < 1e-14)	< .001	0.841	2.02	0	0
3	59	M	N/A	N/A	N/A	N/A	BVH†	< .001	0.04	N/A	0	0
4	51	F	56	Fall, 26, 45	Fall, Fall, 45	158	**	0.118	0.956	14.02	−4	23
5	32	M	46	Fall, Fall, Fall	Fall, Fall, Fall	151	BVH†	< .001	0.514	N/A	0	0
6	45	M	49	Fall, Fall, Fall	Fall, Fall, Fall	130	BVH†	< .001	0.899	N/A	−11	9

Legend.

SOT: Sensory Organization Test. Normal mean composite scores are 80 for 20–59 years olds (yo) & 77 for 60–69 yo. 5th percentile (abnormal) limits are 69 for 20–59 yo & 70 for 60–69 yo. SOT 5 & 6: Average of Sensory Organization Test scores in conditions 5 (eyes closed, sway referenced platform) & 6 (eyes open, both visual surroundings and platform sway referenced). MCT: Motor Control Test. Normal mean composite scores are 143 for 20–59 yo & 152 for 60–69 yo. 5th percentile (abnormal) limits are 161 for 20–59 yo & 171 for 60–69 yo. VOR: Vestibulo-Ocular Reflex, as tested by 50 deg/s peak sinusoidal vertical axis rotation, 0.05 Hz–1 Hz. Midrange gain (0.2 Hz–1 Hz) and time constant estimated with parametric fit to gain & phase data (based on Dimitri et al., 1996 [21]). N/A: not available. *UVH or (pBVH): Unilateral (UVH) or bilateral vestibular (BVH) hypofunction, based on Dimitri et al., 2002 [22]. If patient is scored as BVH, then the probability of this occurring by chance is given in parentheses. RVR: Reduced vestibular response to bilateral, bithermal caloric stimulation. ^†^ Response was too low for accurate estimation of time constant; classified as BVH by low VOR gain and low bilateral ice water calorics. **Classified as abnormal by low scores on CDP SOT 5 & 6.

### Multidirectional vibrotactile feedback system

**Figure 1:**
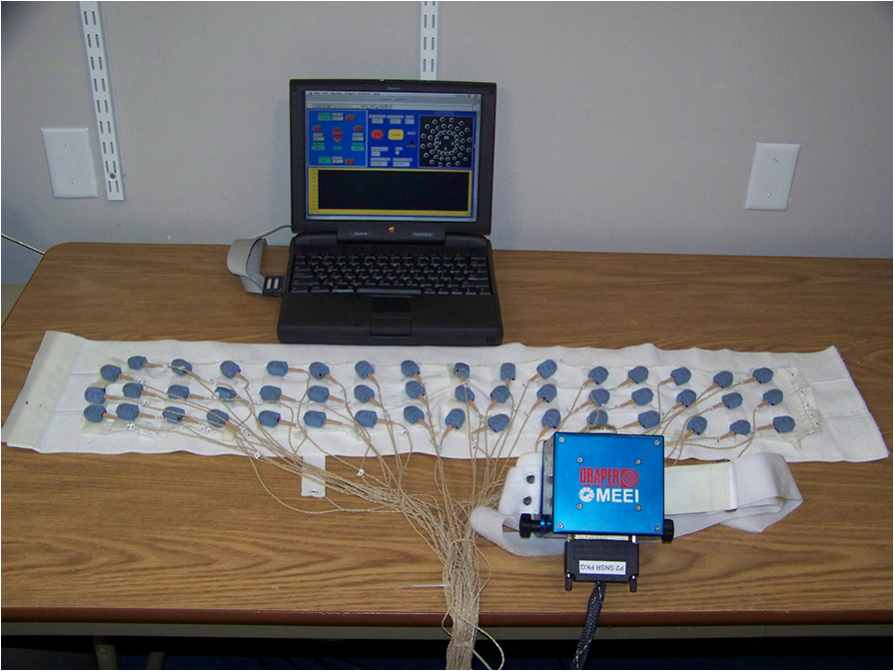
Multidirectional vibrotactile feedback system.

**Figure 2:**
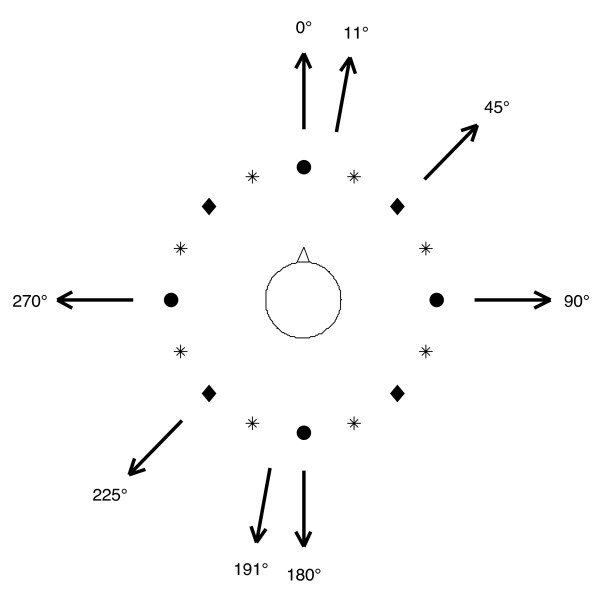
**Vibrotactile display and perturbation directions.** Arrows show the direction of platform motion for the eight discrete perturbations. Subjects were presented vibrotactile feedback using four columns (circles), eight columns (circles and diamonds) or sixteen columns (circles, diamonds, and stars) of tactors.

The multidirectional vibrotactile feedback system [16] consisted of a two-axis inertial measurement unit (IMU) mounted on the lower back of the subject to capture the torso dynamics, a vibrotactile array worn around the torso to intuitively display body motion, and a laptop with analog and digital interfaces (Figure 1). The torso tilt estimates in the A/P and medial-lateral (M/L) directions, referred to as pitch and roll respectively, were obtained by combining the IMU’s accelerometer and gyroscope measurements according to Weinberg, et al., 2006 [18]. The tilt estimates were displayed on a 3-row by 16-column array of tactile vibrators (tactors) worn around the subject’s torso; the rows displayed estimated tilt magnitude and the columns displayed tilt direction. The tilt signal presented to the wearer was a combination of tilt angle and half the tilt rate [18]. A single tactor was activated along the column of tactors that was most closely aligned with the direction of tilt (calculated from the arctangent of the A/P and M/L components) when the displayed tilt exceeded subject-customized preset thresholds. Limits of postural stability were defined by the subject’s maximum static lean while employing an ankle strategy without loss of balance in each of the four cardinal directions during quiet stance. No tactors were activated within the dead zone, a subject-specific zone to allow for normal body sway (0.5° for subject #3, 1° for the others). The lowest row was activated when the tilt exceeded the dead zone threshold. Tactor activation progressed from inferior to superior tactor rows in a stepwise fashion with activation of the middle and highest tactor rows corresponding to a tilt in excess of, respectively, 33% and 67% of the measured limit of stability. Multiple tactor display configurations were evaluated by varying the number of active tactor columns, using 4, 8, or 16 equally spaced columns (Figure 2). In addition, a 4I configuration was treated as two separate single-axis systems, displaying A/P tilt and M/L tilt information independently of each other.

### Protocol

The discrete perturbations, generated by the programmable two-axis Balance Disturber platform (BALDER) [19], perturbed the subjects in the four cardinal directions (0°, 90°, 180° and 270°) which were in alignment with tactor columns present in all display types, two directions (45° and 225°) which were aligned with only the 8 and 16 column displays, and two directions (11° and 191°) which did not coincide with any tactor column among display types (Figure 2). In addition to these eight “testing” directions, the platform moved in six different “training” directions (61°, 155°, 188°, 235°, 267, and 345°) while the subject was learning to use the display. The duration of each perturbation was 400 ms, consisting of a constant platform acceleration for 100 ms, a constant velocity for 200 ms, and a constant deceleration for 100 ms. The perturbation magnitude was determined for each subject according to their abilities by a trial and error process during the training session. Subjects were asked throughout the training session to verbally score the balance difficulty on a scale of 1 to 10, where 10 was defined as the subjects’ “most difficult balance challenge”. The magnitude of the platform motion was adjusted so that balance could be maintained without eliciting a step and the difficulty was rated as 7/10. Perturbation magnitudes ranged from 50 to 70 mm. Two-axis tilt (roll and pitch), center of pressure (COP), and platform position were collected at 100 Hz. The subjects’ feet were positioned in a standard configuration (slightly less than hip-width apart and skewed slightly outward) on the BALDER force plate.

Subjects were first tested with the display turned off, then with each of the four display configurations (collectively referred to as “display on”) in a random order. For each of these four display on trials, subjects were trained on the use of the display, practiced on a one-minute training sequence, and completed a four-minute long testing sequence which included 23 perturbations (ordered to minimize predictability of perturbation direction). A sixth trial was performed with the display off, identical to the first trial but without any additional training. Lastly, a one-minute “erroneous” or sham trial consisted of six perturbations in the testing directions while vibrotactile cues that were typical of the subject’s natural response, but in an unrelated direction, were presented. In order to generate the vibrotactile cues for the sham trials, sway trajectories in response to a unique set of platform perturbations during the training session were recorded (no feedback was provided during this training trial). During the sham trial, the subject received vibrotactile cues consistent with their pre-recorded sway trajectories during the training session; the timing of the perturbations was synchronized, but the directions were unrelated with the sway trajectories. Therefore the feedback did not correlate with the subjects’ actual movements. The erroneous information was displayed using 16 columns of tactors, but is treated separately from the other display on trials.

**Figure 3:**
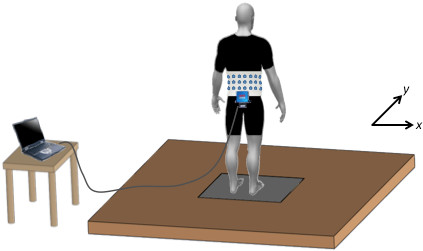
Illustration of experimental set-up.

Subjects were instructed to close their eyes, to keep their arms at their sides, to move to null out the vibrations (i.e. vibrotactile cues were considered “repulsive”, “pushing”, or repellant in nature) and to stand as upright as possible. However, they were not told which tactor configuration they were using unless it was a tactors off trial. Five-minute rest breaks were consistently taken following the completion of two trials. A safety harness was provided and adjusted such that no haptic orientation cues were supplied to the wearer. Figure 3 illustrates the experimental set-up.

### Data analysis

All post-processing and statistics were performed using MATLAB (The MathWorks, Natick, MA). Following data collection, the position and force data from the platform and the tilt data from the IMU were low pass filtered with a 4^th^ order phaseless butterworth filter (MATLAB filtfilt.m) with a corner frequency of 10 Hz to remove high frequency noise, and analyzed for 8 s after each perturbation onset. The trajectories of both the center of pressure data from the platform and the tilt data from the IMU typically showed a three stage response. First, there was a rapid displacement away from baseline until the trajectory reached an extremum in less than one second. Next, there was a return towards the baseline that was then followed by small variations about the baseline. We will refer to these three stages as: “ballistic”, “recovery”, and “steady state” respectively.

**Figure 4:**
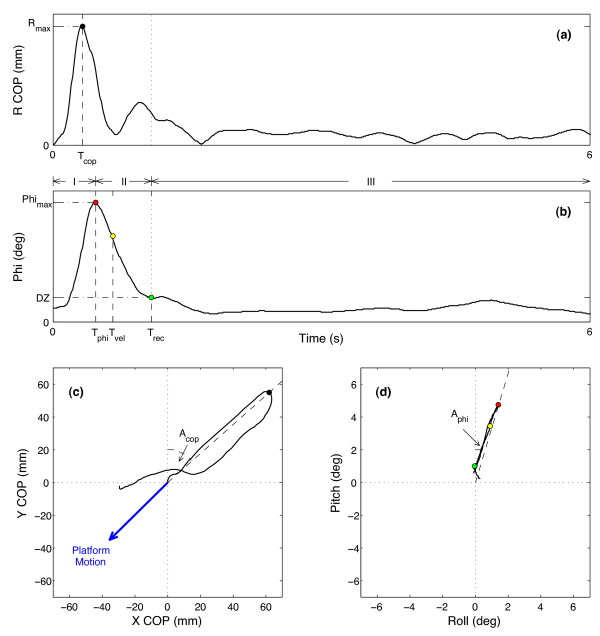
**Sample data from a 225° perturbation.** (**a**) Magnitude of R COP and (**b**) Phi are shown as functions of time, indicating peak values (R_max_, Phi_max_), peak times (T_cop_, T_phi_), recovery time (T_rec_), and time to peak recovery velocity (T_vel_). DZ indicates the degree of tilt within which no tactors are activated. The three stages of the trajectory are marked as I, II, and III for panels (**a**) and (**b**). (**c**) X and Y components of COP are shown in a bird’s-eye view with the peak time (black filled circle) and angle (dashed line), as well as the platform motion (blue arrow). (**d**) A/P and M/L components of tilt are shown in a bird’s-eye view with the peak time (red circle) and angle (dashed line), T_vel_ (yellow filled circle), and T_rec_ (green filled circle).

For each discrete perturbation, X and Y were calculated as the center of pressure in the M/L and A/P directions relative to COP at the start of platform motion (t = 0), and R as the magnitude of the (X,Y) vector. T_cop_ was defined as the time at which R reached its maximum value, R_max_, with A_cop_ as the arctangent of (X,Y) at that time. A_cop_ is measured clockwise from the 12 o’clock position (Figure 4).

Phi was calculated as the magnitude of the resultant (roll, pitch) vector with T_phi_, phi_max_, and A_phi_ extracted similarly to their COP counterparts. Recovery time, T_rec_, was defined as the first time after T_phi_ at which the tilt was within the dead zone, DZ. T_phi_ and T_rec_ partitioned the response into the three abovementioned distinct phases: ballistic, recovery, and steady state. Within the recovery phase, the peak tilt velocity, V_max_, and the time of its occurrence, T_vel_, were determined. The steady state response was parameterized by the percentage of time that tilt was maintained within the dead zone (pct0), and by the RMS of R, phi, and their components, as calculated for five seconds after the recovery time. Parameter values for repeated perturbation directions were averaged within a given trial for a given subject. Statistical tests included a three-way analysis of variance (*anovan.m*) with subject number, perturbation direction, and display as the factor variables, and *post hoc* multiple comparison tests (*multcompare.m*).

## Results

### Response types

A total of 715 perturbations were analyzed across all conditions. Responses were classified into three types on the basis of time to peak tilt: typical (T_phi_ ≤ 1 s), delayed (1 s < T_phi_ ≤ 5 s), and uncontrolled (T_phi_ > 5 s). Typical responses comprised 83% of the data set and exhibited a fast increase (T_phi_ = 0.51 ± 0.09 s) to a single peak, followed by a slower decrease to a steady state (Figure 4). Delayed responses (14%) typically showed two or more peaks, indicating poorer postural control, while uncontrolled responses (3%) exhibited large tilt values after several seconds. Incidences of delayed and uncontrolled responses were significantly reduced (p < .005) with accurate vibrotactile feedback (10.0% and 1.6% respectively) compared to no feedback (18.6% and 2.6% respectively). Erroneous feedback resulted in poorer performance than no feedback (p < .0001) with 30.6% of responses delayed and 22.2% uncontrolled (Table 2). All subsequent analyses were performed only on the typical responses.

**Table 2:** Incidence of response types by display type

	**Display OFF**	**Display ON**	**Erroneous display**
**Typical response**	182	396	17
**Delayed response**	43	45	11
**Uncontrolled response**	6	7	8
Chi-squared value compared to OFF (df = 2)		11.14 (p < .005)	29.24 (p < .0001)

The number of perturbations which resulted in each type of response (typical, delayed, or uncontrolled) is shown for each type of vibrotactile display type (OFF, ON, Erroneous). Chi-squared tests demonstrate significant differences between ON vs. OFF, and between Erroneous vs. OFF.

### Effect of feedback

Overall effectiveness of the vibrotactile feedback was assessed by combining the two trials with the display off, and the four trials with the display on. Mean parameter values across subjects are itemized for each perturbation direction, and then averaged across directions (Table 3). During the ballistic phase, feedback produced only minor differences. R_max_ increased with feedback while T_phi_ decreased (both p < .05). Their counterparts (phi_max_ and T_cop_) showed small, but not statistically significant, changes in the opposite directions and the angles of the peak deflections showed no effect. During the recovery stage, T_vel_ (p < .025) and T_rec_ (p < .0001) were significantly decreased and V_max_ (p < .025) was significantly increased. All steady state tilt parameters exhibited significant improvement with feedback; however, no significant or consistent changes were observed in the steady state COP.

**Table 3:** Significant differences in various parameters due to activation of vibrotactile display

	**Perturbation direction (deg)**									
	**Display**	**0**	**11**	**45**	**90**	**180**	**191**	**225**	**270**	**Mean**
** COP Measures **										
Time to peak deflection (T_cop_, in ms)	OFF	372	361	366	391	369	361	361	388	371
	ON	380	378	367	408	384	361	368	398	381
Mag. of peak deflection (R_max_, in mm)	OFF	67.3	63.0	71.4	83.5	68.6	70.8	78.8	80.8	**73.0**
	ON	68.3	68.8	75.8	84.0	72.7	71.9	79.2	84.7	75.7*
Angle of peak deflection (A_cop_, in deg)	OFF	−179	−166	−125	−92.0	1.16	13.1	50.1	94.1	N/A
	ON	−180	−165	−128	−87.2	−0.17	13.8	49.4	93.7	N/A
RMS of magnitude (R_rms_, in mm)	OFF	14.0	17.9	15.7	20.9	12.9	13.6	16.6	15.6	15.9
	ON	15.4	15.1	14.1	15.4	16.4	16.7	15.1	16.7	15.6
** Tilt Measures **										
Time to peak tilt (T_phi_, in ms)	OFF	576	568	519	153	524	530	488	552	**536**
	ON	512	498	493	520	531	513	491	503	508*
Mag of peak tilt (phi_max_, in deg)	OFF	4.35	4.80	2.99	3.50	7.39	6.41	4.67	3.02	4.64
	ON	4.56	4.03	2.70	2.93	7.15	6.51	4.72	2.85	4.43
Angle of peak deflection (A_phi_, in deg)	OFF	−174	−171	−122	−52.3	−3.90	−0.92	15.6	57.6	N/A
	ON	−174	−169	−126	−60.1	−3.16	−0.77	18.7	81.6	N/A
Max. recovery velocity (V_max_, in deg/s)	OFF	10.5	12.1	6.93	6.31	15.4	13.0	10.7	6.52	**10.2**
	ON	14.6	13.4	7.31	7.13	17.6	14.9	12.6	7.22	**11.9†**
Time of max. velocity (T_vel_, in s)	OFF	0.81	0.76	0.77	1.00	0.73	0.75	0.63	0.95	**0.80**
	ON	0.75	0.70	0.73	0.76	0.74	0.73	0.67	0.72	**0.72†**
Recovery time (T_rec_, in s)	OFF	1.42	2.89	2.19	1.49	3.01	3.09	2.50	1.93	**2.31**
	ON	1.16	1.28	1.49	1.36	1.61	1.43	1.19	1.38	**1.36‡**
RMS of magnitude (phi_rms_, in deg)	OFF	1.05	1.59	1.06	0.86	1.16	1.33	1.04	0.99	**1.13**
	ON	0.92	0.87	0.85	0.77	0.92	1.01	0.83	0.79	**0.87‡**
Pct. of time in dead zone (pct0)	OFF	57.2	45.5	52.6	67.9	49.7	44.1	53.2	57.8	**53.5**
	ON	72.5	76.5	73.7	83.6	73.9	66.5	76.5	79.0	**75.3‡**

* p < .05.

† p < .025.

‡ p < .0001.

Tabulated values are averaged across subjects for each perturbation direction, and then averaged across directions in the final column except for the angles A_cop_ and A_phi_. Levels of significance are determined by a three-way ANOVA with display, subject, and direction as factors.

### Effect of vibrotactile display type

**Figure 5:**
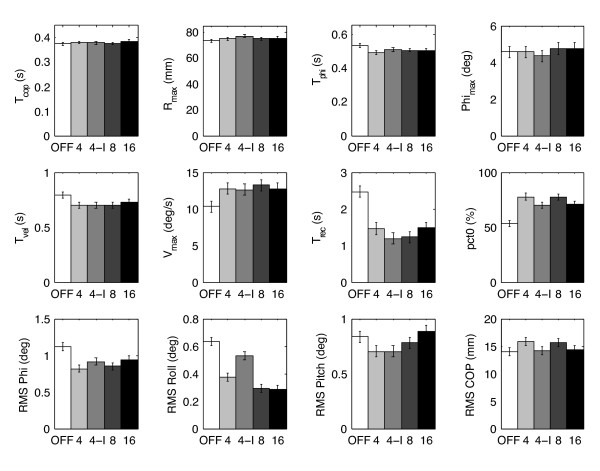
**Variation of parameters (see text for detailed description) across display configurations.** Error bars indicate standard error of the mean. The four configurations with the display on (4, 4-I, 8, 16) show few significant differences amongst themselves, but are each significantly different from the display OFF case for T_phi_, T_vel_, T_rec_, V_max_, pct0, and RMS phi.

Effects due to display type are shown in Figure 5 for the most significant parameters, where the error bars indicate the standard error of the mean. Of the twenty parameters analyzed, spatial resolution exhibited influence on only two: RMS pitch was significantly larger for 16 columns than for the 4 and 4I displays, and RMS roll was significantly larger for the 4I display than any other. RMS phi showed no significant differences among display configurations. For those parameters which showed significant improvements with feedback, the improvements were generally consistent across all display types. In particular, there were no instances where the 4-column display was significantly worse than any other configuration.

### Effects of erroneous feedback

Erroneous feedback produced atypical responses in more than half of the perturbations (19 out of 36). One subject was unable to recover properly from any of the perturbations, while the other subjects showed typical responses between 33% and 100% of the time. Due to the small number of data points, parameter values derived from erroneous feedback were compared to the mean values with the display off for the same combinations of subject and direction, using a *t*-test of paired differences. The only significant difference was an increase in steady state RMS COP (p < .02) with erroneous responses as compared to no feedback.

Similar comparisons were made to values with the display on (accurate feedback). Compared to accurate feedback, erroneous feedback demonstrated significant increases (p < .05) in peak pitch, peak phi, recovery time, RMS pitch, RMS phi, RMS COP and RMS X, and a significant decrease (p < .01) in percentage of time in the dead zone.

### Effects of perturbation direction

**Figure 6:**
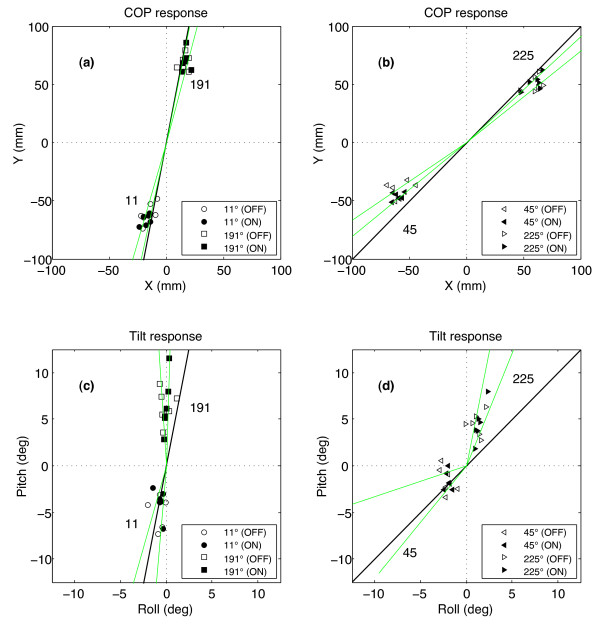
**Peak COP and tilt excursions for non-cardinal perturbation directions.** Mean values are shown for each subject with the display off (open symbol) and on (filled symbol). Green lines indicate 95% confidence intervals of the mean angles (A_cop_ and A_phi_) for each direction and black lines mark the directions of platform motion.

Parameter values during the ballistic phase were highly dependent on the direction of the perturbation. X_max_ and Y_max_ were highly correlated (r^2^ > 0.98) with the M/L component of the direction, and M/L COP deflections were larger than A/P for comparable perturbations (e.g. 90°/270° vs. 0°/180°). For non-cardinal perturbations, this directional asymmetry resulted in a misalignment between A_cop_ and the direction of the perturbation (Figure 6) with A_cop_ being shifted away from the sagittal plane (paired *t*-test, p < .00001), and a significant correlation (r^2^ = 0.77) between R_max_ and the M/L displacement. Tilt parameters showed the opposite effects: phi_max_ correlated with A/P direction (r^2^ = 0.56), roll deflections were much less than pitch, and A_phi_ was shifted towards the sagittal plane for the 191° and 225° perturbations (p < .00001); there was no significant angular shift for the 11° and 45° directions. Paradoxically, peak Y and pitch were both greater for backward perturbations than forward. T_cop_ and T_phi_ were statistically independent of direction. Maximum recovery velocity was also correlated (r^2^ = 0.64, p < .001) with the amount of A/P platform motion. Time to peak velocity and recovery time varied across directions without vibrotactile feedback, but with no consistent pattern; the addition of feedback reduced both the mean times and their variabilities (p < .001).

Steady state parameters showed some significant dependence on direction when no feedback was presented: RMS pitch and phi were larger for A/P perturbations than M/L, and RMS X was larger for M/L. With feedback, the variability across directions was reduced for all steady state parameters and none showed any directional dependencies.

## Discussion

Vibrotactile feedback was found to have the most pronounced effect on subjects’ ability to minimize their body sway and decrease the amount of time spent outside of the dead zone during the steady state phase. This finding is consistent with previously reported studies that demonstrate that individuals with vestibular deficits can use vibrotactile cues during non-perturbed stance with eyes closed [14,15]. Furthermore, the quickened return to upright during the recovery phase when vibrotactile feedback is provided and the minimal impact of feedback during the ballistic phase are consistent with the results obtained by Wall and Kentala. They showed that during A/P perturbations, A/P vibrotactile feedback significantly decreased the peak tilt and the recovery time in subjects with severe postural deficits [15].

While parameters that characterize the recovery and steady state phases of the response trajectory tend to have significant changes when the feedback is on, compared to feedback turned off, we see only small changes in the parameters that characterize the ballistic phase of the trajectory. From this we conclude that the ballistic phase is primarily that of an initial reaction for which there is little time for an active process that depends on motion sensory information to have much of an effect. Based on laboratory-based pilot studies, response times to vibrotactile stimulation applied on the torso can range between 250 and 400 ms (unpublished observations). Given the associated time delays of receiving the vibrotactile sensation, processing the information and responding with an appropriate motor command, it is possible that the subjects have increased the corrective ankle torque, but that the time of peak tilt is too soon to produce an improvement of more than 28 ms. The device becomes more useful, as is evidenced by the discrete perturbation results, during the recovery trajectory and steady state regions as the benefits of the corrections accrue over time. In this case, we observe the effect of the device in terms of significantly faster recoveries, and even more significantly reduced time spent outside of the dead zone and smaller RMS tilt values in the five-second interval following recovery.

Display of erroneous information produced no benefits. Most of the parameters did not differ significantly from those obtained without vibrotactile feedback, and even the small differences were in the direction of poorer performance. Accurate tilt information resulted in significant improvements to several performance metrics, compared to either erroneous, or a lack of, information. Subjects verbally reported an awareness that the erroneous display was not providing useful information, and they tended to disregard the display after a few perturbations. This suggests that subjects are able to interpret the displayed tilt and make corrective maneuvers based upon that interpretation, and that improved postural performance is contingent upon an accurate display.

It has been shown that the hip is the primary means of controlling M/L sway while the ankles are predominantly used to control A/P sway [20]. Because the A/P component of sway dominates instability in natural bipedal stance, it begs the question of whether or not providing information only in that plane would be sufficient for replacing missing vestibular information during surface perturbations. When one more closely examines the physical trajectories of the subjects to the various off-axis perturbations presented in this experiment, one sees that the peak trajectory is not in line with the actual perturbation. Furthermore, the recovery trajectory has a dominant A/P component. This may help explain the recovery behavior we observed of perturbations in non cardinal directions (Figure 6), wherein the peak COP responses tend to shift away from the sagittal plane, while the peak tilt excursions tend to shift towards the sagittal plane. The former shift may well be due to a foot stance in which the M/L width predominates over the A/P one and thus plays a stronger role. It follows that larger restorative torques are exerted in the M/L direction compared to the A/P direction. Thus, the tendency for the body to recover in the opposite direction that shifts toward the sagittal plan could simply be a reaction to that restorative torque.

Limitations to this study include the small sample number, limited number of repetitions that could be performed during the single experimental session, and lack of an age-matched control group. Furthermore, although surface perturbation directions were selected based on their alignment with respect to the activated tactor columns, the resulting body motion did not necessarily follow the same trajectory as the perturbation platform and therefore it is possible that we were not evaluating true off-axis responses.

## Conclusions

Feedback decreases incidences of delayed and uncontrolled responses and produces the greatest effects on body tilt parameters following recovery from the perturbation. Although feedback quickens subjects’ time to return to baseline following a perturbation, the ballistic phase is primarily that of an inertial reaction for which there is little time for feedback to be perceived, processed and acted upon. The findings in this study and in the previous studies that assessed the effect of multidirectional feedback during continuous multidirectional surface perturbations [16,17] suggest that individuals with vestibular deficits are able to use a 4 column display (90° spatial resolution) as effectively as a 16 column display (22.5° spatial resolution) to minimize sway during surface perturbations. From a device design standpoint, less is more: if a simple display provides adequate information regarding torso orientation with respect to the gravito-inertial vector, one should not overengineer the system to provide information that cannot be used to additionally benefit performance [23]. Verbal feedback from the subjects regarding their preference for display type confirmed the quantitative results that there is little difference amongst configurations and that the 4-column display is as good as the 16-column display. These findings are consistent with previously published work by Choweliak et al. regarding the importance of leveraging anchor points (navel, spine, and left and right hand sides) when designing a vibrotactile torso-based display.

## Abbreviations

A/P: Anterior-posterior; BALDER: Balance disturber; COP: Center of pressure; DZ: Dead zone; IMU: Inertial measurement unit; MCT: Motor control test; M/L: Medial-lateral; RMS: Root mean square; SOT: Sensory organization test.

## Competing interests

The authors state that C. Wall is an inventor on an issued patent and has equity interest in BalanceTek, Inc.

## Authors’ contributions

KHS designed the study, carried out the study, analyzed the data, interpreted the data, and drafted the manuscript. MDB developed the experimental instrumentation and software, designed the study, analyzed the data, interpreted the data, and drafted the manuscript. CW conceived of the study, and participated in its design and coordination and helped to draft the manuscript. All authors read and approved the final manuscript.
